# Risk of Endometrial Cancer in Women with Diabetes: A Population-Based Retrospective Cohort Study

**DOI:** 10.3390/jcm10163453

**Published:** 2021-08-04

**Authors:** Lina Zabuliene, Augustė Kaceniene, Laura Steponaviciene, Donata Linkeviciute-Ulinskiene, Rimantas Stukas, Rokas Arlauskas, Rasa Vanseviciute-Petkeviciene, Giedre Smailyte

**Affiliations:** 1 Institute of Clinical Medicine, Faculty of Medicine, Vilnius University, Ciurlionio g. 21, 03101 Vilnius, Lithuania; lina.zabuliene@mf.vu.lt; 2 Laboratory of Cancer Epidemiology, National Cancer Institute, P. Baublio g. 3b, 08406 Vilnius, Lithuania; auguste.kaceniene@nvi.lt; 3 Consultative Polyclinic Department, National Cancer Institute, Santariskių Str. 1, 08660 Vilnius, Lithuania; laura.steponaviciene@nvi.lt (L.S.); rasa.vanseviciute@nvi.lt (R.V.-P.); 4 Institute of Biomedical Sciences, Department of Pathology, Forensic Medicine and Pharmacology, Faculty of Medicine, Vilnius University, Ciurlionio g. 21, 03101 Vilnius, Lithuania; linkeviciutei@gmail.com; 5 Institute of Health Sciences, Faculty of Medicine, Vilnius University, Ciurlionio g. 21, 03101 Vilnius, Lithuania; rimantas.stukas@mf.vu.lt (R.S.); rokas.arlauskas@mf.vu.lt (R.A.); 6 Clinic of Obstetrics and Gynecology, Faculty of Medicine, Institute of Clinical Medicine, Vilnius University, Ciurlionio g. 21, 03101 Vilnius, Lithuania

**Keywords:** endometrial cancer, type 2 diabetes mellitus, glucose-lowering medication, cohort study, cancer risk

## Abstract

The aim of this study was to examine the association between type 2 diabetes (T2DM), use of glucose-lowering medications and endometrial cancer (EC) risk. Methods: The risk of EC incidence among women with T2DM in Lithuania was assessed using a retrospective cohort study design. Female patients who were registered with T2DM between 1 January 2000 and 31 December 2012 were identified in the National Health Insurance Fund database. EC cases (ICD-10 code C54) were identified from the Lithuanian Cancer Registry. Standardized incidence ratios (SIRs) were calculated by dividing the observed numbers of EC among patients with T2DM by the expected number of EC, calculated using national rates. Results: A total of 77,708 diabetic women were included in the analysis, and 995 cases of EC were identified. A significantly increased EC risk in diabetic women was found as compared to the general population (SIR = 1.69, 95% CI 1.59–1.80). The greatest EC risk was found among younger patients at T2DM diagnosis, and the risk declined gradually with increasing age but persisted in being significantly increased among all age groups. The risk for EC increased with increasing duration of diabetes, and the highest EC risk was observed more than 10 years after T2DM diagnosis. A significantly higher EC risk than expected from the general population was found in all patient groups by glucose-lowering medication combinations. The lowest EC risk was observed in diabetic women who were users of “oral only” (without metformin) (SIR = 1.42, 95% CI 1.10–1.83) and “metformin only” (SIR = 1.69, 95% CI 1.49–1.92) medications. A two times greater EC risk was observed among the remaining glucose-lowering medication categories. In contrast, use of insulin only was not related to a higher EC incidence risk (SIR = 0.45, 95% CI 0.23–0.86); however, the risk estimation was based on nine cases. Conclusions: Our study shows a significantly increased EC risk in diabetic women as compared to the general population. In this study, a significantly higher EC risk was found in all patient groups by glucose-lowering medication combinations, except for insulin only users.

## 1. Introduction

Endometrial cancer (EC) is the sixth most common cancer type in women worldwide after breast, colorectal, lung, cervix uteri and thyroid cancers [1]. According to the GLOBOCAN cancer statistics, there were an estimated 417,367 new cases and 97,370 deaths attributed to EC worldwide in 2020 [1]. In recent decades, the incidence and prevalence rates of EC have increased globally [2,3].

Several risk factors have been associated with an increased risk of EC, which include obesity, advancing age, family history of endometrial or colorectal cancer, hereditary predisposition (Lynch syndrome mutations MLH1 or MSH2), early menarche, nulliparity, prolonged unopposed estrogen stimulation and late natural menopause [4,5,6,7,8]. The major risk factors are obesity and diabetes. They share the common pathophysiological basis of hyperinsulinemia and insulin resistance [4,9]. Excess body fat (being overweight or obese), even during childhood and adolescence, is one of the strongest factors that increases the risk for EC [10,11]. Furthermore, insulin resistance leading to hyperinsulinemia is also considered to be a significant risk factor for multiple cancers. In fact, endometrial cells have receptors which bind to insulin with high affinity [12,13,14]. In addition, hyperglycemia caused by type 2 diabetes mellitus, a disease associated with obesity and insulin resistance, is shown to be another independent risk factor for EC and a tumor growth modifier [8].

T2DM is significantly associated with an almost twofold risk of developing EC compared to individuals without diabetes or the general population, and a positive association between diabetes and the risk of EC has been consistently observed independently of geographical region, study design, year of publication and type of diabetes [15,16]. In Lithuania, a higher risk of corpus uteri cancer in diabetic women has also been reported [17]. Some studies have investigated the influence of glucose-lowering medications on EC risk [15,16,18,19,20,21,22,23,24,25], providing contradictory results.

Furthermore, although T2DM rates are continuing to grow globally, there has been important development in the management of this disease and its complications, leading to better long-term outcomes of diabetes patients. Better knowledge of the relationship between diabetes and EC can, hopefully, motivate better prevention and treatment of women with T2DM.

Since the results of previous studies on the effects of glucose-lowering medications on EC risk have been conflicting, there is a need for additional evaluation of the association between the use of glucose-lowering medications and the risk of EC in patients with diabetes. In this study, we examined the association between T2DM, use of glucose-lowering medications and EC risk.

## 2. Materials and Methods

The risk of EC among women with T2DM in Lithuania was assessed using a retrospective cohort study design. Information on diagnosis of T2DM and glucose-lowering medications was obtained from the National Health Insurance Fund (NHIF) database which contains data on prescriptions of reimbursed medications from the year 2000. We analyzed only T2DM cases (International Classification of Diseases (ICD)-10 code E11), diagnosed from age 40, and only patients who had more than 6 prescriptions for reimbursed glucose-lowering drugs were included in the study. EC cases (ICD-10 code C54) were identified from the Lithuanian Cancer Registry, a population-based and nationwide registry that contains personal and demographic data and information on the type of all diagnosed cancer cases in Lithuania, with data from 1978. The records of NHIF were linked to those from the Lithuanian Cancer Registry using the personal identification number. Information about the date of death was obtained from the Causes of Death Register.

Female patients who were registered with T2DM between 1 January 2000 and 31 December 2012 were identified in the NHIF database. Women with cancer diagnosis prior to diabetes diagnosis and women with multiple cancer cases were excluded from the study group. Finally, 77,708 diabetic women were included in the analysis.

Available data for this analysis included: age, date of T2DM onset (diagnosis) and date of EC diagnosis, date of death and prescribed glucose-lowering medications. To assess the T2DM duration impact on EC risk in the study population, the time from T2DM diagnosis was stratified into four groups (<1 year, 1–5 years, 6–10 years and >10 years). Patients with a registered T2DM diagnosis in 2000 include prevalent cases; therefore, they were excluded from the part of analysis involving the duration of T2DM (17,004 cases). EC risk by exposure to glucose-lowering medications was analyzed in seven categories (metformin only; metformin and other oral; oral only (without metformin); insulin only; insulin and other oral (without metformin); metformin and insulin; metformin, other oral and insulin). Women in the exposure group were assigned by glucose-lowering medications used at any time since diabetes diagnosis.

Person-years were computed from the date of T2DM diagnosis to the first of the following events: EC diagnosis, death, emigration or the last follow-up (31 December 2016). For the analysis by exposure to glucose-lowering medications, person-years started 365 days from the date of T2DM diagnosis (1129 cases were excluded). Age and calendar period-standardized incidence ratios (SIRs) were calculated by dividing the observed numbers of EC among patients with T2DM by the expected number of EC, calculated using national rates. Then, 95% confidence intervals (CIs) for SIRs were calculated assuming that the data followed a Poisson distribution. In order to evaluate changes in EC incidence risk of diabetic patients over age groups and T2DM duration, the chi-square (χ^2^) test for trend was performed.

All statistical analyses were carried out using STATA, version 11; StataCorp., College Station, Texas, USA. Excessive adjustment can introduce biases, such as selection bias [26,27]. Directed acyclic graphs (DAGs) provide an entirely graphical yet mathematically rigorous methodology for minimizing bias in epidemiologic studies. The modern theory of diagrams for causal inference and the advantages of using causal models when testing causal associations were recently extensively discussed [28,29]. Potential confounders were selected using directed acyclic graphs (DAGs) based on our a priori knowledge of the relationships among potential confounders and outcome variables [30]. The DAG was created using DAGitty software, version 3.0 (Johannes Textor, Nijmegen, the Netherlands). The study protocol was approved by the Vilnius Regional Biomedical Research Ethics Committee (No. 158200-17-913-423).

## 3. Results

The total follow-up time was 744 807.9 person-years, an average of 9.6 years per subject. In total, 995 cases of EC were identified over the follow-up period, instead of the expected 587, showing a significantly increased EC risk in women with T2DM (SIR = 1.69, 95% CI 1.59–1.80) as compared to the general population (Table 1). The greatest EC risk was among younger patients at T2DM diagnosis (SIR = 2.29, 95% CI 1.86–2.81). The SIRs declined gradually with increasing age (test for trend *p* < 0.001) but persisted in being significantly increased among patients with T2DM aged 70 and older (SIR = 1.32, 95% CI 1.14–1.52).

During the first year after T2DM diagnosis, the EC risk was found to be significantly lower in diabetic patients than in the general population (SIR = 0.07, 95% CI 0.02–0.21); however, the result was based on three cases of EC. The risk of EC increased with increasing time since diagnosis (test for trend *p* < 0.001), and the highest disadvantage in EC risk was observed more than 10 years after T2DM diagnosis (SIR = 2.07, 95% CI 1.72–2.48).

With regard to treatment, a significantly higher EC risk than expected from the general population was found in all patient groups by glucose-lowering medication combinations, except for insulin only users (Table 2). From these groups, the lowest EC risk was observed in diabetic women who were users of oral only (without metformin) (SIR = 1.42, 95% CI 1.10–1.83) and metformin only (SIR = 1.69, 95% CI 1.49–1.92) medications. Women treated with all types of glucose-lowering medication combinations (metformin/other oral/insulin) during the follow-up period had the highest risk of EC development (SIR = 2.46, 95% CI 1.99–3.05). Additionally, a two times greater EC risk was observed among the remaining glucose-lowering medication categories. In contrast, use of insulin only was inversely related to the EC incidence risk (SIR = 0.45, 95% CI 0.23–0.86).

**Figure 1:**
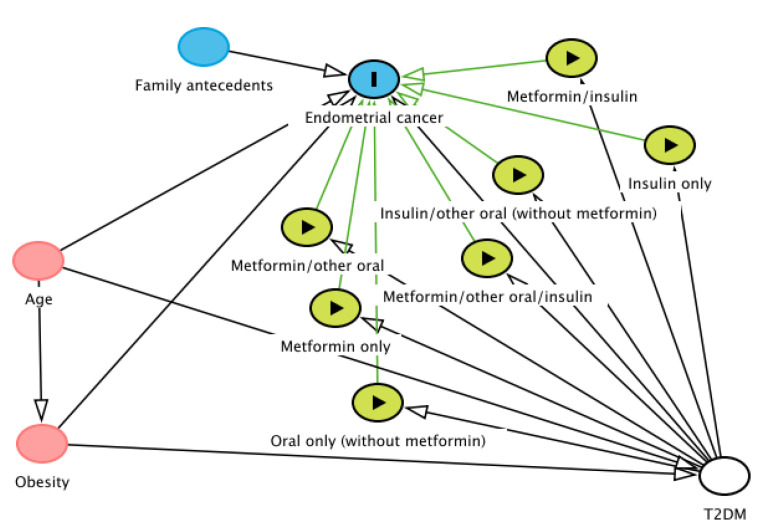
Directed acyclic graph (DAG) showing causal assumptions on the effect of glucose-lowering medications (exposure) on endometrial cancer (outcome)*. * Adjusted variable (white color), ancestors of exposure and outcome (red color), exposures (green color), ancestor of outcome (blue color). Based on DAGitty version 3.0 (Johannes Textor, Nijmegen, the Netherlands).

DAG analysis showed that for the evaluation of the effect of treatments in endometrial cancer, the only variable to control is T2DM (Figure 1). In our study, we controlled it by design, restricting it to only diabetic patients.

## 4. Discussion

Our study showed a significantly increased EC risk in diabetic women as compared to the general population. The greatest EC risk was found among younger patients at T2DM diagnosis, and the risk declined gradually with increasing age but persisted in being significantly increased among all age groups. The risk for EC increased with increasing duration of diabetes, and the highest EC risk was observed more than 10 years after T2DM diagnosis.

Earlier studies, such as a meta-analysis by Zhang et al., have shown that there is a significantly higher risk of EC associated with T2DM [15]. A larger systemic review and meta-analysis by Liao et al. confirmed these findings [16]. It is calculated that nearly 40% of the worldwide burden of EC cases is attributable to the combination of diabetes and obesity [31]. Both T2DM and EC are diseases that are age-associated and share common risk factors; thus, the chance to develop T2DM and EC might be increasing in today’s aging society [16].

In our study, a significantly higher EC risk than expected from the general population was found in all glucose-lowering medication combinations groups, except for insulin only users. Various epidemiological studies have shown that metformin use may be associated with a lower risk for [20,25] and better survival from cancer [21]. Metformin is the first-line medication worldwide for the treatment of T2DM, which acts by reducing glucose production and stimulating glucose uptake in muscle cells, thus lowering circulating glucose and insulin levels [32]. The direct mechanisms of metformin mainly include inhibition of the cellular energy-sensing liver kinase B1-AMP-activated protein kinase and PI3K-Akt-mammalian target of rapamycin inhibition (mTOR) and insulin-like growth factor 1-related signaling pathways, which reduces the proliferation and promotes the apoptosis of EC cells [33]. Metformin interferes with key immunopathological mechanisms that are involved in pathological processes or associated with malignant progression [34]. It is suggested that the antiproliferative effect of metformin in therapeutic doses is due to indirect mechanisms, lowering of hyperglycemia, insulin and insulin resistance, IGF-1 and leptin levels, and it also decreases chronic inflammation and increases the blood levels of sex hormone binding globulin, which leads to reductions in circulating estrogen and androgens [33,35].

Several meta-analyses have shown inconsistent results on the association between metformin treatment and EC risk [19,22,23,36]. A pooled analysis of five studies found that metformin use was associated with a 13% reduction in EC risk among patients with diabetes (RR = 0.87, 95% CI 0.80–0.95) [23]. The pooled results of seven studies suggested that metformin use is neutral for the risk of EC (OR = 1.05, 95% CI 0.82–1.35) [22]. In contrast, another meta-analysis of over 500 thousand patients, of which 300 thousand received metformin treatment, found a higher EC incidence in the metformin use group (OR = 1.29, 95% CI 1.16–1.44) [23].

Our study did not show greater advantages of metformin use on EC risk, as also reported in the following studies [22,31,37]. Chu et al. did not find a change in EC risk among metformin users, compared to other treatments (OR = 0.99, 95% CI 0.78–1.26) [22]. Similarly, neither ever-use nor long-term use (≥25 prescriptions) of metformin were shown to have an effect on EC risk in a study by Becker et al. [37]. In contrast, a retrospective analysis of over 92 thousand women, of which 590 were diagnosed with EC, showed a higher risk of EC in metformin ever-users both in the full cohort (HR = 1.23 95% CI 1.03–1.48) and the case–control analysis (HR = 1.24, 95% CI 1.02–1.51) [36]. In addition, a higher cumulative dose showed an increasing risk of EC, and there were no differences in EC risk between metformin and other glucose-lowering medication use.

Results of other studies [38,39] have suggested that, among glucose-lowering medications, insulin and sulfonylureas, drugs that increase insulin secretion, may increase the cancer risk through the interaction with insulin and IGF-1 receptors, leading to stimulation of cell proliferation [35,40]. In our study, use of insulin only was inversely related to the EC incidence risk (SIR = 0.45, 95% CI 0.23–0.86); however, the risk estimation was based on nine cases. Therefore, it is too soon to draw any definite conclusions based on this small group. Furthermore, since insulin only is not the usual treatment for T2DM treatment, these patients might have had specific clinical conditions where this type of treatment was indicated, such as misdiagnosed type 1 diabetes, secondary insulin insufficiency or end-stage organ failure.

The meta-analysis by Tian et al. showed that use of insulin (as compared with non-use in patients who were diagnosed with diabetes but did not use insulin) was not associated with a significant increase in the risk of EC in patients with diabetes in three observational studies (OR = 1.15, 95% CI 0.93–1.40) [23]. In a study from Italy by Franchi et al., including 376 diabetic women with endometrial cancer and 7485 diabetic controls matched for cases on age, date at cohort entry and duration of follow-up, no significant associations with EC were observed for insulin at cohort entry (OR = 0.72, 95% CI 0.34–1.56) and at follow-up (OR = 1.19, 95% CI 0.82–1.71). The authors did not find significant associations with EC risk and use of sulfonylureas at cohort entry (OR = 1.14, 95% CI 0.91–1.42) or at follow-up (OR = 1.16, 95% CI 0.91–1.47) either [41]. On the other hand, Arima et al. found that “ever-use” of insulin was associated with an increased incidence of EC compared to never use, and, furthermore, a trend towards an elevated EC risk was seen with increasing cumulative use of insulin [36]. That study also showed that “ever-use” of other forms of oral glucose-lowering medications (mostly sulfonylureas) was associated with an increased incidence of EC compared to never use. In contrast, Becker et al. found that long-term use of sulfonylureas was not associated with the risk of EC (adjusted OR = 0.96, 95% CI 0.65–1.44) [37]. Similar to our results, in a nested case–control study using data from the British Columbia Cancer Registry, women receiving multiple types of medications over a long time had the highest risk of EC [42].

To sum up, the results of various studies on the probable impact of glucose-lowering medications are, indeed, conflicting. After analysis of our findings, based on the higher risk of EC with a longer T2DM duration and a younger T2DM onset, as well as the higher risk when combinations with several oral glucose-lowering medications, with or without insulin, were used, we hypothesize that in diabetic women, the extent of insulin resistance and the severity of T2DM, rather than the effect of any specific medication, may be associated with EC risk.

This study has several strengths and limitations. The strengths lie in the long duration of follow-up and the large national population-based cohort. Additionally, this study assessed the possible effect of all of the most commonly used glucose-lowering medication combinations on EC risk in women with T2DM throughout the whole observation period and not only at study entry. Furthermore, the information was acquired from well-established registries and not from questionnaires. However, since data were collected retrospectively, we could not evaluate the importance of confounding factors such as body mass index, obesity, smoking history, lifestyle, dietary habits, family history, pregnancies, parity and menopause status or directly measure clinical parameters such as insulin levels or insulin resistance; therefore, the linkage between hyperinsulinemia and cancer incidence could only be inferred from the medication–cancer associations. Lastly, the results might be affected by the varying patterns of glucose-lowering medication use over time.

## 5. Conclusions

Our study shows a significantly increased EC risk in diabetic women as compared to the general population. In this study, a significantly higher EC risk was found in all patient groups by glucose-lowering medication combinations, except for insulin users.

## Figures and Tables

**Table 1:** EC risk by age and time after diagnosis among women with T2DM in Lithuania.

	Obs ^1^	Exp ^2^	SIR ^3^	95% CI	
Overall	995	587.19	1.69	1.59	1.80
Age at diagnosis					
40–49	89	38.92	2.29	1.86	2.81
50–59	331	173.43	1.91	1.71	2.13
60–69	391	235.08	1.66	1.51	1.84
≥70	184	139.76	1.32	1.14	1.52
	Trend: χ^2^ = 434.4; *p* < 0.001				
Time after T2DM diagnosis ^4^					
<1	3	43.42	0.07	0.02	0.21
1–5	329	172.09	1.91	1.72	2.13
6–10	244	147.42	1.66	1.46	1.88
>10	114	55.15	2.07	1.72	2.48
	Trend: χ^2^ = 475.3; *p* < 0.001				

^1^ observed; ^2^ expected; ^3^ standardized incidence ratio; ^4^ excluded diabetes cases registered in 2000.

**Table 2:** EC risk by glucose-lowering medications among women with T2DM in Lithuania.

	Obs ^1^	Exp ^2^	SIR ^3^	95% CI	
Overall	992	532.14	1.86	1.75	1.98
Glucose-lowering medication					
Metformin only	239	141.44	1.69	1.49	1.92
Metformin/other oral	532	263.00	2.02	1.86	2.20
Oral only (without metformin)	59	41.50	1.42	1.10	1.83
Insulin only	9	20.19	0.45	0.23	0.86
Insulin/other oral (without metformin)	11	5.30	2.08	1.15	3.75
Metformin/insulin	58	26.60	2.18	1.69	2.82
Metformin/other oral/insulin	84	34.12	2.46	1.99	3.05

^1^ observed; ^2^ expected; ^3^ standardized incidence ratio.

## Data Availability

The dataset used during the current study is available from the corresponding author on reasonable request.
